# *PpNAC1*, a main regulator of phenylalanine biosynthesis and utilization in maritime pine

**DOI:** 10.1111/pbi.12854

**Published:** 2017-11-23

**Authors:** María Belén Pascual, María‐Teresa Llebrés, Blanca Craven‐Bartle, Rafael A. Cañas, Francisco M. Cánovas, Concepción Ávila

**Affiliations:** ^1^ Departamento de Biología Molecular y Bioquímica Facultad de Ciencias Universidad de Málaga Campus Universitario de Teatinos Málaga Spain

**Keywords:** maritime pine, *MYB*, phenylalanine biosynthesis and utilization, *PpNAC1*, transcription factor, regulator

## Abstract

The transcriptional regulation of phenylalanine metabolism is particularly important in conifers, long‐lived species that use large amounts of carbon in wood. Here, we show that the *Pinus pinaster* transcription factor, *PpNAC1,* is a main regulator of phenylalanine biosynthesis and utilization. A phylogenetic analysis classified PpNAC1 in the NST proteins group and was selected for functional characterization. *PpNAC1* is predominantly expressed in the secondary xylem and compression wood of adult trees. Silencing of *PpNAC1* in *P. pinaster* results in the alteration of stem vascular radial patterning and the down‐regulation of several genes associated with cell wall biogenesis and secondary metabolism. Furthermore, transactivation and EMSA analyses showed that *PpNAC1* is able to activate its own expression and *PpMyb4* promoter, while PpMyb4 is able to activate *PpMyb8*, a transcriptional regulator of phenylalanine and lignin biosynthesis in maritime pine. Together, these results suggest that *PpNAC1* is a functional ortholog of the *Arabidopsis* SND1 and NST1 genes and support the idea that key regulators governing secondary cell wall formation could be conserved between gymnosperms and angiosperms. Understanding the molecular switches controlling wood formation is of paramount importance for fundamental tree biology and paves the way for applications in conifer biotechnology.

## Introduction

Conifers like other woody plant species irreversibly immobilize large quantities of carbon skeletons in wood during their long life cycles. Particularly in these species, the metabolism of phenylalanine, precursor amino acid for lignin, is essential for secondary cell wall biosynthesis and must be finely regulated at transcriptional level (Pascual *et al*., 2016). To elucidate the transcriptional network, controlling wood formation in conifers is of paramount importance for future applications in fundamental tree biology and biotechnology.

Several transcription factors (TF) regulating the biosynthesis of secondary cell wall components have been described in poplar (Sterky *et al*., 2004), eucalyptus (Goicoechea *et al*., 2005; Rengel *et al*., 2009), white spruce (Pavy *et al*., 2005) and pine (Allona *et al*., 1998; Bedon *et al*., 2007; Craven‐Bartle *et al*., 2013; Gómez‐Maldonado *et al*., 2004; Lorenz and Dean, 2002; Patzlaff *et al*., 2003a,b; Villalobos *et al*., 2012). Focusing on the transcriptional network, the best wood‐associated transcription factors characterized belong to the MYB and NAC families. Both transcription factor families are the most represented in plants with over 100 members in Arabidopsis, rice and poplar (Hu *et al*., 2010; Martin and Paz‐Ares, 1997; Nuruzzaman *et al*., 2010; Ooka *et al*., 2003). However, the NAC TF family in conifers is underrepresented with over 37 members (Pascual *et al*., 2015).

In *P. taeda,* PtMyb1, PtMyb4 and PtMyb8 activate transcription of genes involved in phenylpropanoid and lignin biosynthesis through the binding to AC elements present in their promoter regions (Craven‐Bartle *et al*., 2013; Gómez‐Maldonado *et al*., 2004; Patzlaff *et al*., 2003a,b). In addition, the overexpression of these Myb TFs led to ectopic lignin deposition producing plants with an increased secondary wall thickening (Bomal *et al*., 2008; Patzlaff *et al*., 2003a,b).

A functional role in wood formation has been shown for some NAC proteins (Yamaguchi and Demura, 2010). In *Arabidopsis,* the NAC protein subfamily with the capacity to induce cell wall secondary biosynthesis has been named the VNS family, and some members of this family such as NST1 (secondary wall thickening promoting factor 1), SND1 (secondary wall‐associated NAC domain protein 1), VND6 (vascular related NAC domain 6) and VND7 act together as key regulators along the entire secondary cell wall biosynthesis programme (Mitsuda *et al*., 2007; Zhong *et al*., 2006) and activate a cascade of downstream TFs involving Myb proteins. A similar transcriptional network involving NAC proteins known as WNDs (wood‐associated NAC domain TFs) and Mybs has been described to operate in poplar and eucalyptus (Zhong and Ye, 2009; Zhong *et al*., 2011). In *Arabidopsis*, Myb46 and Myb83, targets of SND1, are key regulators of the biosynthesis of cellulose, hemicellulose and lignin, three major secondary cell wall components (Zhong *et al*., 2007a). Several wood‐associated Mybs, such as EgMyb2 from eucalyptus, PtrMyb83 and PtrMyb20 from poplar, and PtMyb4 from pine, are functional orthologs of *Arabidopsis* Myb46 and Myb83 (Goicoechea *et al*., 2005; McCarthy *et al*., 2010; Patzlaff *et al*., 2003a,b). Furthermore, in a previous study, we showed that PpMyb8 from maritime pine regulates phenylpropanoid metabolism (Craven‐Bartle *et al*., 2013). These data suggest that PtMyb4 and PtMyb8/PpMyb8 may be members of a transcriptional cascade controlling lignin biosynthesis in conifers (Bomal *et al*., 2008).

In this report, we have identified and characterized *PpNAC1* from *P. pinaster,* a potential ortholog of the *Arabidopsis* NST1 and SND1 genes that is expressed in the xylem and compression wood of adult trees. We have found that silencing of *PpNAC1* alters the morphology of *P. pinaster* plantlets, which exhibit delayed growth, thickened hypocotyls and a disorganized vascular structure. Furthermore, we have seen that *PpNAC1* is able to activate its own expression and that of the *PpMyb4* transcription factor, which in turn controls the expression of *PpMyb8*. Altogether, our findings suggest that *PpNAC1* and the downstream transcription factors, *PtMyb4* and *PtMyb8,* are involved in a transcriptional regulatory network controlling phenylalanine metabolism in maritime pine.

## Results

### Isolation and characterization of *PpNAC1* from *P. pinaster*

**Figure 1:**
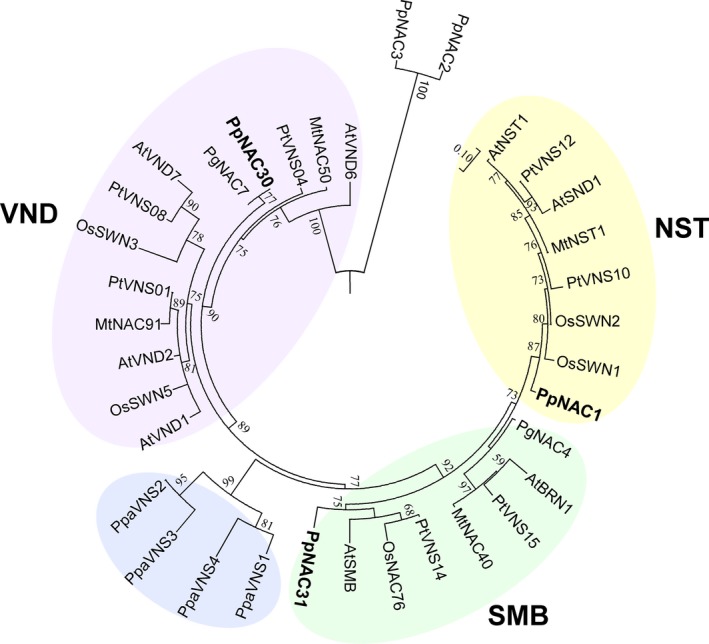
Phylogenetic analysis of full‐length VNS proteins. Bootstrapping was performed with 1000 replicates. The VNS proteins are classified into three principal groups: VND, NST and SMB. Pp, *Pinus pinaster*; At, *Arabidopsis thaliana*; Os, *Oryza sativa*; Pt, *Populus trichocarpa*; Pg, *Picea glauca*; Mt, *Medicago truncatula*; Ppa, *Physcomitrella patens*. Percentage bootstrap values no less than 50% are presented. The accession numbers and sequences of the NAC proteins used in the analysis are available in Table S2.

We have previously identified 37 genes that encode NAC transcription factors in the *P. pinaster* genome (Pascual *et al*., 2015). The NAC TFs involved in vascular development has been referred as the VNS family (Ohtani *et al*., 2011; Xu *et al*., 2014) and their members are divided into three groups namely, VND, NST and SMB, by phylogenetic analysis. In *P. pinaster,* we have identified three *PpVNS*‐type genes: *PpNAC1* belonging to the NST group, *PpNAC30* in the VND group and *PpNAC31* of the SMB group (Figure 1). Considering that no VNS genes belonging to the NST group have been identified in gymnosperms, we selected *PpNAC1* for a further functional characterization.

**Figure 2:**
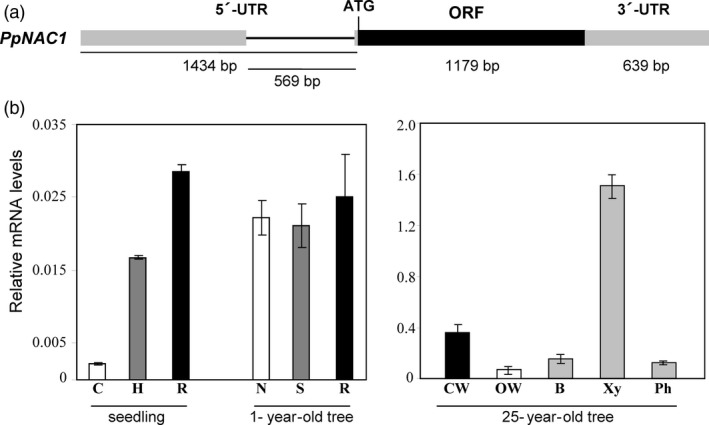
*PpNAC1* cDNA structure and expression analysis. (a) Diagram of the *PpNAC1* cDNA. The coding region of the gene is represented by black boxes; the grey boxes represent untranslated regions and the thinner black line in the 5′‐untranslated region represents an intron. The length in base pairs of each region is also shown. (b) Expression of *PpNAC1*. Transcript levels were determined in different organs of *P. pinaster* by qPCR using specific primers (Table S1). The expression data were normalized using *Actin* and *EF1‐alpha* as reference genes. Data are mean standard deviation from three biological replicates. C, cotyledons; H, hypocotyl; R, root; N, needles; S, stem; CW, compression wood; OW, opposite wood; B, bark; Xy, secondary xylem; Ph, phloem.

Using the sequence information available within the *P. pinaster* (http://www.scbi.uma.es/sustainpinedb/sessions/new) and *P. taeda* (https://dendrome.ucdavis.edu/resources/databases/) databases, the cDNA of *PpNAC1* was cloned using PCR and fully sequenced. The full‐length cDNA of *PpNAC1* consists of 3252 bp, including a 1434‐bp 5′‐untranslated region (5′‐UTR), an open‐reading frame (ORF) of 1179‐bp encoding a protein of 392 amino acid residues and a 639‐bp 3′‐untranslated region (3′‐UTR). Using genomic sequence information available in *P. pinaster,* we were able to observe that this gene contains an intron of 569 bp inside the 5′UTR (Figure 2a).

### Expression profiling of *PpNAC1*

The expression pattern of *PpNAC1* gene was analysed by qPCR in different samples from maritime pine seedlings, 1‐year‐old and 25‐year‐old trees (Figure 2b). Transcripts were detected in all samples examined, but their relative levels were particularly high in tissues undergoing secondary wall thickening such as the roots and hypocotyls of seedlings and the needles, stems and roots of 1‐year‐old trees. Expression analysis in laser‐microdissected samples from pine seedling cell types (Cañas *et al*., 2017) revealed that *PpNAC1* was highly expressed in root developing cortex, a tissue with active cell wall biosynthesis (Figure S1). However, the highest mRNA level of *PpNAC1* (50‐ to 100‐fold of those detected in seedlings) was found in secondary xylem from 25‐year‐old trees. Furthermore, the transcript accumulation of *PpNAC1* was fivefold higher in compression than in the opposite wood (Figure 2b).

### *PpNAC1*_RNAi lines

**Figure 3:**
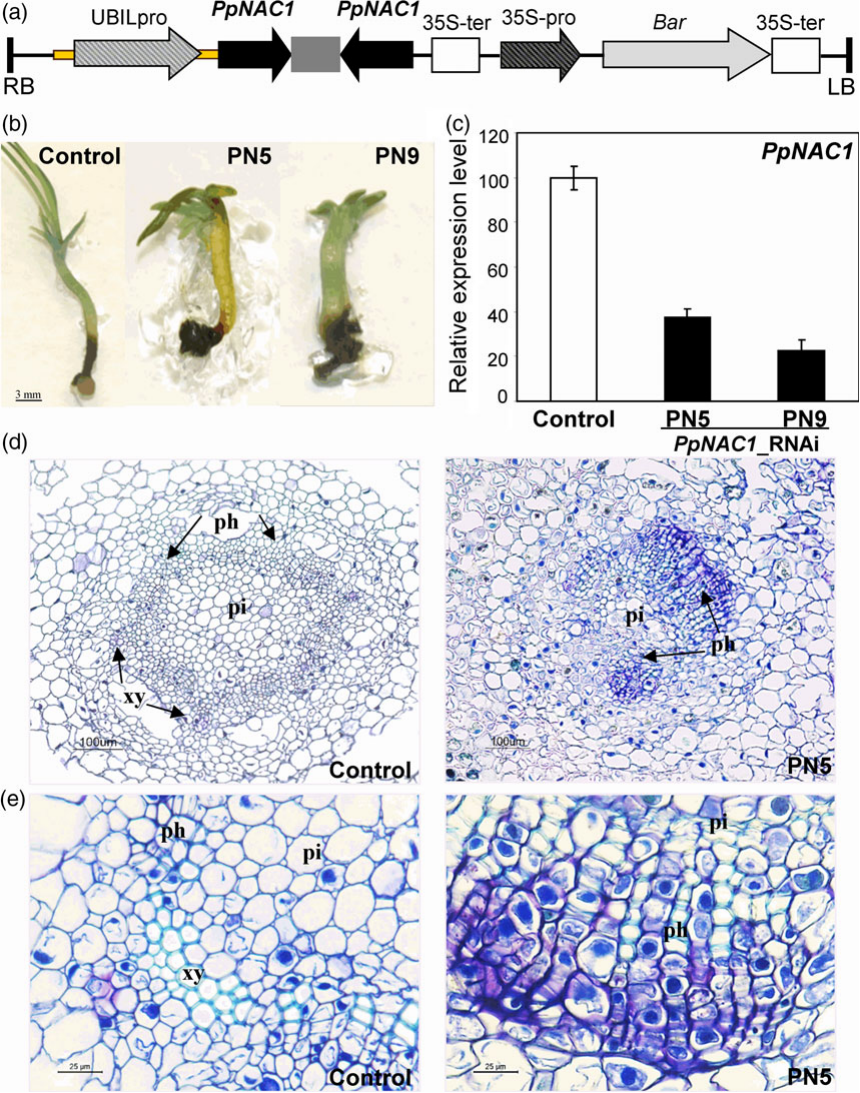
*PpNAC1*_RNAi lines in *P. pinaster*. (a) Diagram of the construct used for the silencing of *PpNAC1*. The black arrow represents the fragment of 400 bp used for gene silencing which expression is driving by the maize UBIL promoter. A grey box between black arrows is a spacer. This vector harbours a BASTA (gen *bar*) selectable marker under the direction of cauliflower mosaic virus 35S gene promoter (CaMV35). (b) Phenotypes of untransformed PN519 (control line)) and two independent *PpNAC1* silencing transgenic lines (PN5 and PN9) after 60 days *in vitro* germination in MLV medium. (c) qPCR analysis of *PpNAC1* expression in control (white) and PN5 and PN9 (black) plantlets. Data are mean standard deviation from four biological replicates, each comprised by pooling tissue from three plantlets. Data were normalized to *EF1‐alpha* as a reference gene. Expression levels are shown as the percentage of the value obtained for wild‐type plants (100%). (d, e) Cross sections (10 μm) in the hypocotyl of 60‐d‐old *in vitro* germinated plantlets. A phloem zone expanded with great number of cells can be observed in PN5 section. The histological sections were stained with Toluidine blue. Ph, phloem; xy, xylem; pi, pith.

To functionally characterize *PpNAC1* in *P. pinaster*,* PpNAC1*_RNAi lines were generated via somatic embryogenesis using a hairpin construct. We selected a specific fragment of 400 bp of the *PpNAC1* gene to avoid the down‐regulation of other related targets and cloned it into the gateway vector pBb7GW‐I‐WG‐UBIL. This vector incorporates a BASTA selectable marker and the maize UBIL promoter to drive the expression of the transgene (Figure 3a). Ten independent RNAi transgenic lines were obtained, and the presence of the transgene was confirmed by PCR. The plantlets germinated for 60 days had thickened hypocotyls and poor growth compared with untransformed PN519 plants (Figure 3b). Moreover, their growth was severely delayed, particularly the PN5 and PN9 lines. *PpNAC1* transcript levels were considerably lower in all silenced lines than in control plants, and the two independent lines, PN5 and PN9, showing the highest reduction in *PpNAC1* expression, were selected for further analyses (Figure 3c). We also examined the effects of RNAi inhibition of *PpNAC1* expression on the hypocotyl structure (Figure 3d, e). Cross sections of the stems showed that the vascular morphology of the RNAi plants had a slightly disorganized stem, with a phloem zone expanded with great number of cells and with an altered vascular radial patterning when compared with untransformed PN519 plants.

### Transcriptome expression profiling of *PpNAC1_*RNAi *P. pinaster* plantlets

**Figure 4:**
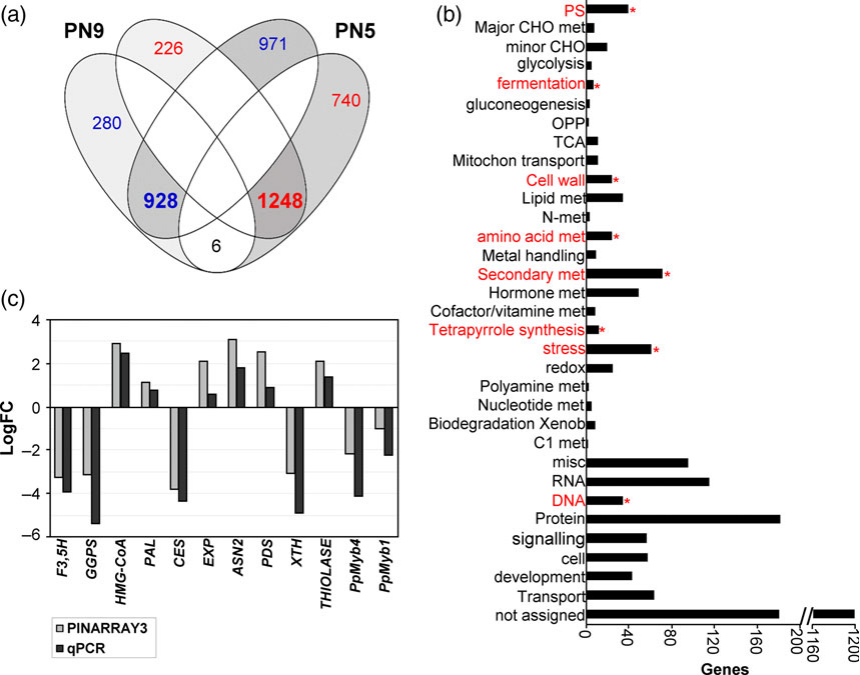
Transcriptome analysis of untransformed PN519 (control) and *PpNAC1*_RNAi plantlets. (a) Venn diagram showing both unique and overlapping (bold) expressed genes of significantly up‐regulated (blue) and down‐regulated (red) genes between PN5 and PN9 RNAi transgenic plants. (b) Functional enrichment analysis of the functional categories. The horizontal bars represent the number of genes included in each functional category. Functional categories and asterisks in red show significant different categories between the *PpNAC1*_RNAi and control samples with a *P*‐value < 0.05 using the Fisher's exact test. (c) Validation of microarray results by qPCR. Fold changes (LogFC) of gene expression in control and *PpNAC1*_RNAi lines (mean of PN5 and PN9) samples analysed using PINARRAY3 and qPCR are shown. Positive values correspond to higher expression in *PpNAC1_* RNAi samples and negative values to higher expression in control samples. F3,5H, Flavonoid 3′,5′‐hydroxylase; GGPS, geranylgeranyl pyrophosphate synthetase; HMG‐CoA, 3‐hydroxy‐3‐methylglutaryl‐coenzyme A reductase; PAL, phenylalanine ammonia‐lyase; CES, cellulose synthase; EXP, expansion gene; ASN2, asparagine synthetase 2; PDS, phytoene desaturase; XTH, xyloglucan endotransglucosylase/hydrolase; THIOLASE, thiolase family protein; PpMyb4, *P. pinaster* Myb4 transcription factor; PpMyb8, *P. pinaster* Myb8 transcription factor.

To determine whether the down‐regulation of the *PpNAC1 gene* in *P. pinaster* plantlets resulted in large changes in gene expression, the transcriptomes of silenced and PN519 control plantlets were compared. Total RNA was isolated from PN519, PN5 and PN9 plantlets germinated for 60 days, and the transcriptomes were analysed using a 4x44K custom array (PINARRAY3). Genes with an adjusted *P*‐value below 0.05 and a logarithm fold‐change in expression of 0.5 or greater were considered differentially expressed between the transgenic and control lines. For the PN5 line, the number of up‐ and down‐regulated transcripts was 1899 and 1994, respectively. For the PN9 line, the numbers of up‐ and down‐regulated transcripts were 1214 and 1474, respectively. For transcriptomic analysis, only genes having differential expression levels in both transgenic lines were considered (Figure 4a; Table S3). Using this criterion, the down‐regulation of *PpNAC1* had a strong effect on the pine transcriptome with 928 up‐regulated and 1248 down‐regulated common genes in both lines (Figure 4a). Functional annotations indicated that many differentially expressed genes were related to cell wall biogenesis, amino acid metabolism and secondary metabolism (phenylpropanoids, flavonoids and terpenoids) (Figure 4b; Table S4). Key genes for enzymes involved in the monolignol biosynthesis, such as *p*‐coumarate 3‐hydroxylase (*C3H*), shikimate *O*‐hydroxycinnamoyltranferase (*HCT*), caffeoyl‐CoA O‐methyltransferase (*CCoAOMT*) and cinnamyl‐alcohol dehydrogenase (*CAD*), were down‐regulated in the transgenic plants. Reduced transcript levels were observed for cellulose synthase *(CESA4),* xyloglucan endotransglucosylase/hydrolase *(XTH)* and laccase (*LAC*) genes involved in secondary cell wall biosynthesis and reassembly. Genes encoding α‐tubulins and putative microtubule‐associated proteins (MAPs) were also down‐regulated in the RNAi plants. Other genes involved in flavonoid and isoprenoid biosynthesis, such as flavonoid 3′,5′‐hydroxylase (*F3*′*5*′*H*), geranylgeranyl pyrophosphate synthetase (*GGPS*) and chalcone synthase (*CHS*), were also strongly down‐regulated in the transgenic lines. Additionally, transcription factors such as *PpMyb1, PpMyb4* and *PpMyb8* were down‐regulated in the RNAi plantlets (Figure S2). The validation of the microarray data was performed by qPCR analysis of 12 differentially expressed genes. Figure 4c shows a comparison of the transcript levels determined by microarray and qPCR analyses, and the results were similar.

### Isolation of *PpNAC1*,* PpMyb4* and *PpMyb8* promoters and *in silico* analysis of putative cis elements

**Figure 5:**
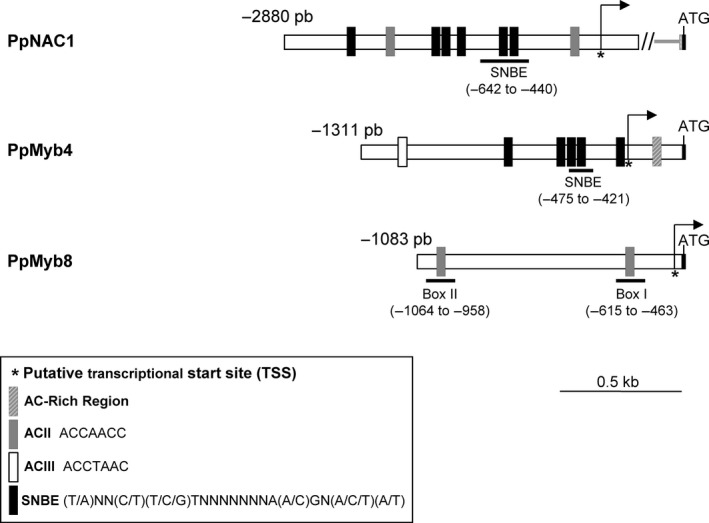
Distribution of SNBE and AC elements in the *PpNAC1*,* Myb4* and *Myb8* promoters. In the schematic representation of the promoters, the position of the transcriptional start site is indicated (arrow). The number to the left of each promoter indicates the relative distance to ATG. The presence of SNBE (black rectangles) and different class AC elements (grey and white rectangles) are shown. An AC‐rich region in the *Myb4* promoter is indicated as striped rectangle. The elements used for further analysis are underlined, and their positions relative to the ATG start codon are indicated.

NAC proteins bind to *cis*‐acting element containing a consensus sequence of 19 bp named secondary wall NAC binding element (SNBE). This motif is present in many promoters in the *Arabidopsis* genome (Wang *et al*., 2011), including the promoter of Myb46, which is a direct target of SND1 (Zhong *et al*., 2006, 2007a, 2010c). We have isolated the corresponding promoter region of *PpNAC1* (KY451900)*,* which is 1446 pb in length (Figure 5).

Simultaneously, and considering that microarray data pointed to a possible coordinated expression of *PpNAC1* and MYB transcription factors regulating lignin biosynthesis, the 5′ flanking regions of *PpMyb4* (KY451898) and *PpMyb8* (KY451899) were isolated and contained 1311‐pb and 1083‐pb upstream of the ATG, respectively. Figure 5 shows a diagram representing the promoter region for the *PpNAC1*,* PpMyb4* and *PpMyb8* genes and putative *cis* elements identified using PLACE database (http://www.dna.affrc.go.jp/PLACE/). In the *PpNAC1* promoter, at least six putative SNBEs were found as well as one AC element of the AC‐II class. In the *PpMyb4* promoter, five putative SNBE sites, one canonical AC element of the AC‐III class in the distal region and an AC‐rich region (spanning a sequence of 20 bp) proximal to the ATG were found. The upstream region of the *PpMyb8* contained two AC elements of the AC‐II class (Box I and Box II) and none SNBE element (Figure 5).

### PpNAC1 protein binds to the SNBE element present on its own promoter to self‐activation of gene expression

**Figure 6:**
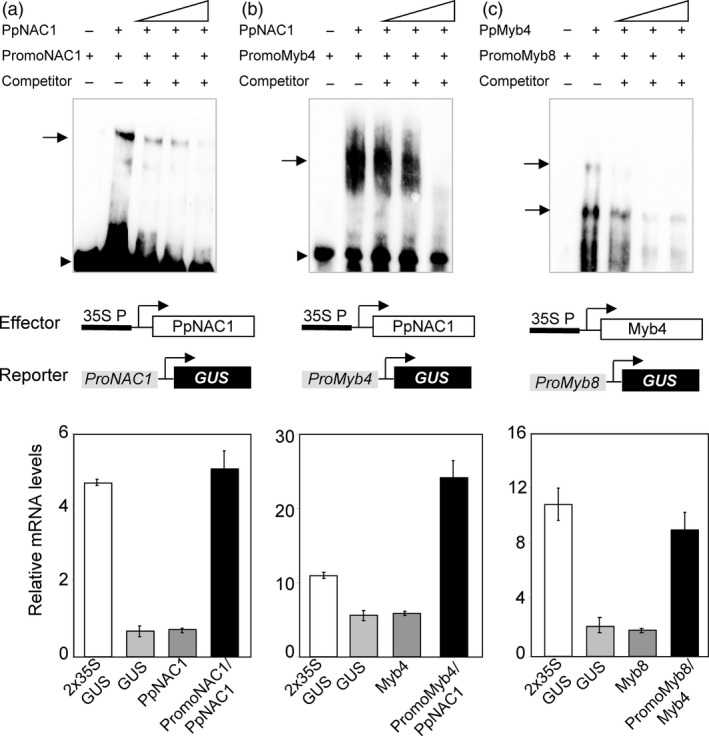
EMSA and transactivation analyses. (a) PpNAC1, (b) PpMyb4 and (c) PpMyb8. In the upper panels, EMSA shows that PpNAC1 binds to their own promoter and to a region in the *Myb4* promoter that contains SNBE sites. The purified protein Myb4 binds to a portion of the *Myb8* promoter that contains AC elements. No band shift was seen in the controls without the addition of protein. Unlabelled promoter fragments in 10‐, 25‐ and 50‐fold molar excess relative to the labelled probes were used as competitors. Arrows represent shifted complexes. In the lower panels, diagrams of the reporter and effector constructs and transactivation analysis. The effector construct contains the CaMV 35S promoter‐driven PpNAC1 and PpMyb4. In each reporter construct, the *GUS* gene was driven by the corresponding promoter: *PpNAC1, PpMyb4* and *PpMyb8*. Transactivation analyses show the PpNAC1‐mediated transcriptional activation of *PpNAC1* (a) and *PpMyb4* (b), and the PpMyb4‐mediated transcriptional activation of *PpMyb8* (c). The *GUS* RNA levels were assayed in pine stem protoplasts co‐transformed with a combination of reporter and effector plasmids at a 1:1 molar ratio. Protoplasts transfected with the reporter or effector construct alone were used as negative controls and the *GUS* RNA levels driven by the 35S promoter were used of positive control (white bar). Bars represent ± SDs from three biological replicates.

Previous works have described that SND1 of *Arabidopsis* regulates their own expression, binding directly to an SNBE motif present in its own promoter. To address this possibility in the regulation of *PpNAC1,* we investigated whether PpNAC1 was able to bind its own promoter using electrophoretic mobility shift assays (EMSA). For this analysis, we cloned a 200‐pb promoter fragment by PCR containing three putative SNBE motifs present in the upstream region of the gene (from −440 pb to −642 pb, Figure 5). The gel shift observed with PpNAC1 protein was abolished by adding a competitor DNA proving to be specific (Figure 6a, upper panel). Furthermore, in the transactivation assay using *P. pinaster* protoplasts, the *PpNAC1* promoter was activated approximately fivefold when the protoplasts were cotransfected with the effector construct 35S:PpNAC1 (Figure 6a, lower panel). These results indicate that, as occurs in *Arabidopsis* for SND1, the PpNAC1 protein can bind directly to its own promoter and activates its expression.

### PpNAC1 protein binds to the SNBE element present on the *PpMyb4* promoter and activates its expression

In *Arabidopsis,* it has been previously shown that SND1 is able to bind and transactivate the *Myb46* promoter (Zhong *et al*., 2007a). To examine a possible binding of PpNAC1 to the *PpMyb4* promoter, protein–DNA interactions were analysed using EMSA. PpNAC1 protein was able to bind at a portion of the promoter that contains a SNBE site (from −421 pb to −475 pb, Figure 5) in the *PpMyb4* promoter causing a clearly visible mobility shift (Figure 6b, upper panel). The complex formed was efficiently competed by the addition of the unlabelled SNBE *Myb4* promoter confirming the specificity of the formed complex. Next, we tested the possible functional significance of the formation of this complex in *P. pinaster* protoplasts. For this assay, pine stem protoplasts were cotransfected with two constructs: the effector construct containing PpNAC1 driven by a tandem duplication of the cauliflower mosaic virus (CaMV) 35S promoter and the reporter construct containing the *GUS* reporter driven by the *PpMyb4* promoter. As shown in Figure 6b (lower panel), PpNAC1 enhanced *GUS* expression under the *PpMyb4* promoter by approximately fivefold the levels observed in the absence of the TF.

### PpMyb4 protein binds to the AC‐II element present on the PpMyb8 promoter and activates its expression

Previous studies have supported a role of PpMyb8 in the regulation of the phenylalanine pathway (Craven‐Bartle *et al*., 2013). *In silico* analysis revealed the presence of two AC‐II class elements in the *PpMyb8* promoter: Box I (from −463 to −615 pb) and Box II (from −958 to −1064 pb) (Figure 5). To test a possible transcriptional control of *PpMyb8* by PpMyb4, EMSA and transactivation analyses were performed. Both *in vitro* and *in vivo* assays (Figure 6c, upper and lower panels) demonstrated a direct interaction of Myb4 protein with the *PpMyb8* promoter resulting in the transcriptional activation of *PpMyb8* by approximately fourfold the levels observed in the absence of the TF (Figure 6c, lower panel).

## Discussion

### *PpNAC1* is a potential ortholog of the *NST* genes of angiosperms

The identification of the molecular switches that regulate secondary cell wall biogenesis during wood formation is essential for basic studies and also for the biotechnological manipulation of wood quality and quantity in woody plant species.

Functional studies with wood related NAC TF of *Arabidopsis* such as NST1/2, SND1/2, VND proteins and SOMBRERO (SMB) and BEARSKIN1/2 proteins, have indicated that they are key transcriptional regulators of secondary cell wall (SCW) biosynthesis and have been classified into VND, NST or SMB groups by phylogenetic analysis (Nakano *et al*., 2015). So far no direct correlation has been found between the number of VNS genes present in a plant species and the size of its genome or the abundance of lignified tissues (Nakano *et al*., 2015; Zhu *et al*., 2012). For example, the moss *P. patens* contains eight VNS genes in its genome (Xu *et al*., 2014; Zhu *et al*., 2012), while *P. trichocarpa* and *E. grandis*, two woody angiosperms, have 16 (Ohtani *et al*., 2011; Zhong *et al*., 2010b) and six VNS genes (Hussey *et al*., 2015; Myburg *et al*., 2014), respectively. In conifers, *P. abies* has four (Nystedt *et al*., 2013), *P. glauca* two (Duval *et al*., 2014) and *P. pinaster* three VNS genes (Pascual *et al*., 2015).

The phylogenetic analysis of VNS genes showed that *P. pinaster* presents one gene classified in each group (Figure 1) and points to *PpNAC1* as a potential ortholog of the *SND1* gene of *Arabidopsis*. To our knowledge, no members of the NST group have been previously identified in gymnosperms. In *P. glauca,* only two VNS genes have been identified; *PgNAC7* is a VND‐type gene functionally similar to the *AtVND6* and its expression is preferentially associated with vascular tissue in the stem, while the *PgNAC4* clustered with the SMB group, and its expression was clearly predominant in root tips (Duval *et al*., 2014), as described in *Arabidopsis* (Ohtani *et al*., 2011; Zhong *et al*., 2010b). *PpNAC1* is predominantly expressed in the secondary xylem and compression wood of adult trees, tissues undergoing lignin biosynthesis (Figure 2b). This transcript level distribution is consistent with previous observations reported for the PtrWNDs in *Populus* and the SND1, NSTs and VNDs in *Arabidopsis* (Kubo *et al*., 2005; Zhong and Ye, 2010; Zhong *et al*., 2007b).

Although the NAC family in conifers (37 putative members in *P. pinaster)* appears to be underrepresented when compared with the more than 100 members in *Arabidopsis* or poplar, the identification of *PpNAC1* suggests that the primary layer of the NAC master switch for secondary cell wall formation has been evolutionarily conserved in vascular plants.

### *PpNAC1*_RNAi lines exhibit altered vascular differentiation

One common strategy for functional characterization of a candidate gene is to down‐ or up‐regulate its expression by genetic transformation. The long generation time and long life span of conifers have been major obstacles to perform reverse genetic approaches in these woody plants, and the functional studies of many conifer genes have been performed in *Arabidopsis* and tobacco (Newman *et al*., 2004; Patzlaff *et al*., 2003b). Nevertheless, advances have been made in the generation of transgenic conifers via somatic embryogenesis, and efficient protocols are currently available for genetic transformation and cryopreservation of embryogenic cell lines and subsequent plant regeneration (Klimaszewska *et al*., 2004; Trontin *et al*., 2007). Using these protocols, we have generated RNAi *P. pinaster* lines for *PpNAC1*. The morphological phenotypes, vascular tissue architecture and expression analysis of RNAi_*PpNAC1* plantlets strongly suggest that *PpNAC1* is associated with vascular development (Figures 3 and 4). In *Arabidopsis*, down‐regulation of both SND1 and NST1 genes resulted in loss of secondary cell wall in the xylem fibres of stem and consequently in a lower stem strength. In addition, several genes involved in the secondary wall biosynthesis were down‐regulated in these plants (Zhong *et al*., 2007b). The pine transcriptome was also strongly disturbed in the *PpNAC1*_RNAi plantlets, resulting in altered expression of a range of genes implicated in cell wall biogenesis, amino acid metabolism and secondary metabolism (Figure 4; Tables S3 and S4). Key genes for enzymes of monolignol biosynthesis, such as *C3H*,* CAD* or *CCoAOMT*, were down‐regulated in the transgenic plants. In *P. radiata* has been shown that CCoAOMT is needed for biosynthesis of guaiacyl lignin and its suppression modifies lignin content and composition resulting in a lignin polymer with an unusual subunit composition (Wagner *et al*., 2011). Reduced transcript levels were also observed for *CesA4* and *XTH,* which are involved in secondary cell wall biosynthesis and reassembly during growth and differentiation. XTH modifies xyloglucan, the major hemicellulose present in the primary cell walls of pine trees (Valenzuela *et al*., 2014). Laccase genes were also down‐regulated in the RNAi plants. Suppression of *LAC4* and *LAC17* expression in *Arabidopsis* affected lignin biosynthesis mainly in fibre cells of the inflorescence stem (Berthet *et al*., 2011; Schuetz *et al*., 2014; Zhao *et al*., 2013).

Genes encoding α‐tubulins and putative microtubule‐associated proteins (MAPs) were also down‐regulated in the pine RNAi plants. In *Arabidopsis,* the silencing of AtMAP70‐5 protein produces atrophied plants exhibiting disorganized vascular elements, suggesting that these proteins are essential for secondary cell wall biogenesis and for the adequate development of xylem (Pesquet *et al*., 2010).

A remarkable finding is that *PpMyb1*,* PpMyb4* and *PpMyb8* genes were down‐regulated in the RNAi plantlets (Figure S2). These *Myb* genes are expressed in secondary xylem and have been functionally associated with phenylpropanoid and lignin biosynthesis in *P. glauca* (Bedon *et al*., 2010; Bomal *et al*., 2008), *P. pinaster* (Craven‐Bartle *et al*., 2013) and *P. taeda* (Patzlaff *et al*., 2003a,b).

### A transcriptional regulation network controlling phenylpropanoid biosynthesis in maritime pine

It is well known that some NAC proteins (SND1, NST1 and VNDs) act as master regulators of a signalling cascade that involves R2R3‐MYBs and regulates vascular development and secondary cell wall formation in *Arabidopsis*. Functional orthologs of this network have been identified in poplar (Lin *et al*., 2013; Zhong *et al*., 2013) and eucalyptus, but functional studies in conifers are scant. Duval *et al*. (2014) reported that *PgNAC7* could be a master regulator of secondary cell wall biosynthesis in conifer xylem. Recently, it has been proposed that *PgNAC8* could also regulate cellulose biosynthesis in coordination with *PgNAC7* (Lamara *et al*., 2016). Phylogenetic and expression analyses of *PgNAC8* have suggested a role as a potential candidate ortholog of SND2/3 genes regulating complex carbohydrate biosynthesis (Lamara *et al*., 2016; Zhong *et al*., 2010a).

The *in vitro* transactivation and EMSA analysis showed that PpNAC1 can bind directly to its own promoter to activate transcription through a positive feedback loop (Figure 6a). Likewise, PpNAC1 was able to activate *PpMyb4* expression (Figure 6b), while PpMyb4 was able to activate *PpMyb8* (Figure 6c). Furthermore, the transcript levels of *PpMyb4* and *PpMyb8* were drastically reduced when *PpNAC1* was silenced in the transgenic plants (Figure S2).

**Figure 7:**
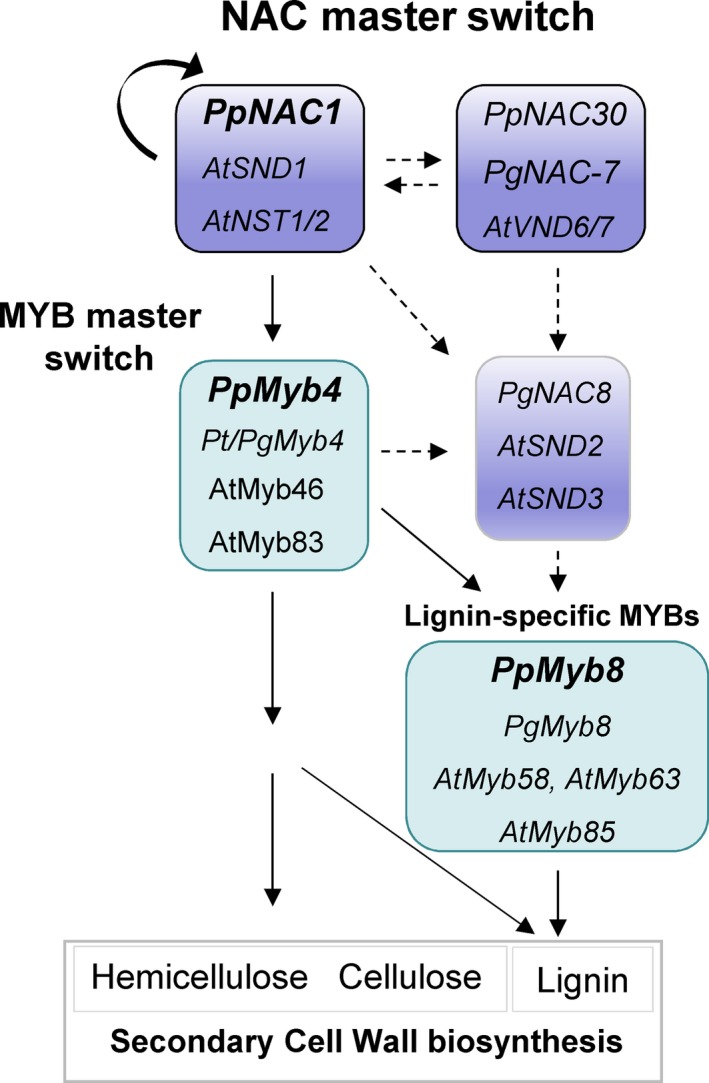
Proposed model of transcriptional regulatory network controlling secondary cell wall biosynthesis in *P. pinaster*. Transcription factors from *Arabidopsis*,* P. pinaster* and *P. glauca* that have been functionally characterized in the network are shown. Continuous lines indicate that the interactions have been functionally demonstrated, while the dashed lines indicate interactions that have not yet been demonstrated.

The results obtained in this work suggested that a transcriptional cascade similar to the SND1 network defined in *Arabidopsis* (Zhong *et al*., 2006) and poplar (Lin *et al*., 2013; Wang *et al*., 2014) is conserved in conifers (Figure 7). To gain insight into this question, we have generated Arabidopsis plants overexpressing PpNAC1. The overexpression of PpNAC1 produced a prominent phenotypic effect in Arabidopsis plants with small rosette size and curled leaves (Figure S3a). In addition, an up‐regulation of secondary wall biosynthetic genes was observed (Figure S3b) together with increased transcript levels of secondary cell wall‐associated TFs (Figure S3c). This behaviour is similar to that of previously described in Arabidopsis plants overexpressing SND1, NST1, PtrWND2B and PtrWND6B (Mitsuda *et al*., 2005; Zhong *et al*., 2006, 2010a,b,c).

Moreover, Myb58 and Myb63 transcription factors, regulated by SND1 and Myb46 in Arabidopsis, in turn regulate the expression of genes involved in lignin biosynthesis (Zhou *et al*., 2009). These MYB factors are significantly up‐regulated in the PpNAC1 overexpressing Arabidopsis plants, strongly suggesting a functional role of PpNAC1 as ortholog of the Arabidopsis SND1 transcription factor (Figure S3).

PpNAC1 may be a regulator that could act at the first level of transcriptional control in phenylalanine metabolism to promote wood formation, while PpMyb4 would function upstream of PpMyb8 and other MYBs.

In conifers, PpMyb8 and PgMyb8 are involved in the coordinated expression of lignin biosynthesis through binding to *cis* regulatory elements present in the promoter region of key genes in the pathway (Bomal *et al*., 2014; Craven‐Bartle *et al*., 2013). Moreover, transgenic plants overexpressing *PtMyb8* showed up‐regulation of genes encoding phenylpropanoid enzymes and misregulation of several cell wall‐related genes (Bomal *et al*., 2008). PpMyb4 and its orthologs, PtMyb4 and EgMyb2, could be nonspecific for regulating lignin biosynthesis because they are also involved in the regulation of cellulose and xylan biosynthesis (Zhong *et al*., 2013). Taken together, the results presented here suggest that PpNAC1, PpMyb4 and PpMyb8 are activators of lignin biosynthesis in maritime pine. The identification of *PpNAC1* as a main regulator of this network involved in wood formation in *P. pinaster* is of great interest for fundamental studies in conifers but also for potential applications in tree biotechnology. To increase our knowledge about the transcriptional regulatory network operating in conifers, and given the complexity of the network demonstrated in other species, intensive research is necessary to fully clarify to what extent the transcriptional network could be conserved between gymnosperms and angiosperms.

## Experimental procedures

### Plant material

*Pinus pinaster* Ait. seeds were supplied by the Centro de Recursos Genéticos Forestales “El Serranillo” (Ministerio de Agricultura, Pesca, Alimentación y Medio Ambiente, Spain). The seed germination and growth of the seedlings were performed as described elsewhere (Pascual *et al*., 2015). Cotyledons, hypocotyls and roots from pine seedlings (1‐month‐old) and needles, stems and roots from young trees (1‐year‐old) were collected separately, frozen in liquid nitrogen and stored at −80 °C until use. Samples of bark, xylem, phloem, and compression and opposite wood were collected from maritime pine 25‐year‐old trees of Sierra Bermeja (Estepona, Spain) (Villalobos *et al*., 2012).

### Constructs and pine transformation

The full‐length *PpNAC1* cDNA was cloned from pine seedling hypocotyl RNA by PCR amplification of the specific sequence in the *P. pinaster* database (http://www.scbi.uma.es/sustainpinedb/sessions/new).

To obtain RNA interference (RNAi), we used a hairpin construct. For this, a fragment of 400 bp from the *PpNAC1* gene was amplified using specific Gateway primers, cloned into pDONR207 (Invitrogen, Germany) and introduced into the monocot‐specific vector pBb7GW‐I‐WG‐UBIL, which contains a BASTA selectable marker and makes use of maize ubiquitin (UBIL) promoter to drive the expression of the transgene. The *A. tumefaciens* strain C58C1 was transformed by electroporation.

The *P. pinaster* embryogenic cell line PN519 (Trontin *et al*., 2007) has been used and maintained according to Klimaszewska *et al*. (2001). The transformation of PN519 was performed as previously described (Klimaszewska *et al*., 2004) and transferred to proliferation medium containing plant growth regulators. For the maturation of control and transformed embryos, tissues were transferred to maturation medium supplemented with abscisic acid (ABA). The mature embryos were germinated for 2 months in controlled conditions. Genomic DNA was isolated from Basta‐resistant embryonal tissues and transgenics confirmed by PCR analysis. Independent transgenic lines exhibiting reduced levels of *PpNAC1* transcripts were selected for embryo maturation and production of somatic embryo plants. In addition, the untransformed PN519 line was used as a control. Primers used are listed in Table S1.

### Plantlets growth and histology

Somatic embryos were germinated for 60 days on MLV medium contained 87 mM sucrose. *In vitro* plantlets were frozen into liquid nitrogen and stored at −80 °C until use. For histological analysis, hypocotyls were immediately fixed in 4% (v/v) paraformaldehyde in 0.1 M phosphate buffer (pH 7.4) under vacuum (three times for 15 min). The samples were dehydrated and infiltrated with paraffin for 5 days. Thin sections (10 μm) were prepared using a microtome, and paraffin‐free sections were stained with 1% Toluidine blue.

### Isolation of the promoter regions of *PpNAC1*,* PpMyb4* and *PpMyb8*

Isolation of genomic DNA was performed using the CTAB method (Doyle and Doyle, 1987), and the promoter sequence of *PpNAC1* was obtained by PCR amplification using primers designed from the loblolly pine database (https://dendrome.ucdavis.edu/resources/databases/). The promoters of *PpMyb4* and *PpMyb8* genes were obtained by PCR walking. A list of primers is provided in Table S1.

### RNA isolation and qPCR

The isolation of RNA was performed as described elsewhere (Canales *et al*., 2012). RQ1 RNase‐Free DNase (Promega Corporation, Madison, WI) was used for the removal genomic DNA contamination from RNA samples, and cDNA synthesis was performed with iScript Reverse Transcription Supermix (Bio‐Rad^®^). Real‐time PCR (qPCR) was performed according to Canales *et al*. (2012). *Actin* and *elongation factor‐1‐alpha* (*EF1‐*α) were used as reference genes. The gene‐specific primers used are listed in Table S1.

The laser capture microdissection procedure and qPCR analysis were carried out as described (Cañas *et al*., 2017).

### Microarray hybridization

Somatic embryos were germinated for 60 days on MLV medium contained 87 mM sucrose. Two RNAi transgenic lines, PN5 and PN9, as well as the control cell line PN519, were used, with three biological replicates per line and six somatic embryo plants per replicate.

A custom microarray (PINARRAY3) was used that includes 60‐mer oligonucleotides designed using the *P. pinaster* transcriptome (Canales *et al*., 2014). Slides were made by Agilent Technologies, and hybridization was performed at 65 °C following the protocol described by Cañas *et al*. (2015). Then, the slides were washed and air‐dried. Hybridized slides were scanned, and signal intensities were recorded. The differentially expressed genes were identified using the Limma package for R (Smyth, 2005). The microarray data are accessible at NCBI′s Gene Expression Omnibus (Edgar *et al*., 2002) through the accession number GSE89341.

### Protein expression and EMSA

The full‐length cDNAs of *PpNAC1* and *PpMyb4* were amplified and cloned into the pDEST17 vector (Invitrogen, Germany). The production of the recombinants proteins was induced in the *E. coli* strain BL21‐AI at 20 °C for 5 h in presence of 0.2% arabinose. The recombinant PpNAC1 and PpMyb4 proteins were purified by affinity chromatography and used for EMSA with the *PpNAC1, PpMyb4* and *PpMyb8* promoter fragments.

The primers used to amplify the promoter DNA fragments were labelled with biotin at the 5′ terminus, and their sequences are provided in Table S1. For EMSAs, 1 μg of purified PpNAC1 or PpMyb4 was incubated at room temperature for 30 min with the biotin‐labelled promoter fragment in the binding buffer (10 mm Tris, pH 7.5, 5 mm MgCl_2_, 2.5% glycerol, 0.05% NP‐40, 100 ng/μL poly(dI‐dC)). For competition analysis, unlabelled fragments were added in the reactions as competitors in a 10‐, 25‐ or 50‐fold molar excess relative to the labelled probes. The samples were resolved in 5% polyacrylamide nondenaturing gel, electrotransferred onto nylon membranes and signals revealed using a chemiluminescence kit (Thermo Fisher scientific).

### Transient expression analysis in pine protoplasts

The procedure was performed following the protocol described previously (Gómez‐Maldonado *et al*., 2004). To prepare the reporter constructs, the promoter sequences of *PpNAC1*,* PpMyb4* and *PpMYB8* were cloned into the pBI221 plasmid replacing the CaMV 35S promoter. Each of the reporter constructs was co‐transformed with the corresponding effector construct into pine stem protoplasts according to Gómez‐Maldonado *et al*. (2004). After incubation for 16 h in dark, the protoplasts were retrieved by centrifugation at 500 ***g*** for 3 min and frozen in liquid nitrogen. The GUS RNA levels were determined using specific primers presented in Table S1.

### Maximum‐likelihood phylogenetic analysis

The phylogenetic analysis was conducted with 35 full‐length sequences from seven species: *Pinus pinaster*,* Picea glauca*,* Populus trichocarpa*,* Arabidopsis thaliana*,* Medicago truncatula* and *Oryza sativa*. Four *Physcomitrella patents* NAC proteins were used as outgroup to root the tree. Multiple alignments were carried out using MUSCLE v3.8.31 (Edgar, 2004), and tree topology was inferred using maximum likelihood with PhyML (Guindon and Gascuel, 2003; Guindon *et al*., 2005). The bootstrap test was carried out with 1000 replicates. The MEGA 7.0 software (Kumar *et al*., 2016) was used to draw phylogenetic trees. The accession numbers of the NAC sequences are available in Table S2.

## Supporting information


**Figure S1** Expression profile of *PpNAC1* in laser‐microdissected tissues from one‐month‐old *P. pinaster* seedlings.
**Figure S2** qPCR analysis of *PpMyb1, PpMyb4* and *PpMyb8* expression in control (white) and *PpNAC1*_RNAi (mean of PN5 and PN9, grey) plantlets.
**Figure S3** Expression analysis of genes and transcription factors involved in the biosynthesis of secondary cell wall components in PpNAC1 overexpressing Arabidopsis plants.

**Table S1** Oligonucleotides used in this work.

**Table S2** Names, gene accession numbers and sequences of the NAC protein used in the phylogenetic analysis.

**Table S3** Microarray results. Up‐regulated differential expressed genes are highlighted in red. Down‐regulated differential expressed genes are highlighted in blue.

**Table S4** Functional enrichment analysis results.
