# Supplementary material for: PpNAC1, a main regulator of phenylalanine biosynthesis and utilization in maritime pine
Source: Plant Biotechnol J. 2017 Nov 23;16(5):1094–104. doi: 10.1111/pbi.12854 (PMC5902770; doi:10.1111/pbi.12854)
Supplement: Supplementary file 1 — Figure S1 Expression profile of PpNAC1 in laser‐microdissected tissues from one‐month‐old P. pinaster seedlings. Figure S2 qPCR analysis of PpMyb1, PpMyb4 and PpMyb8 expression in control (white) and PpNAC1_RNAi (mean of PN5 and PN9, grey) plantlets. Figure S3 Expression analysis of genes and transcription factors involved in the biosynthesis of secondary cell wall components in PpNAC1 overexpressing Arabidopsis plants. [file PBI-16-1094-s004.docx]

**Supporting information**

**Figure S1**


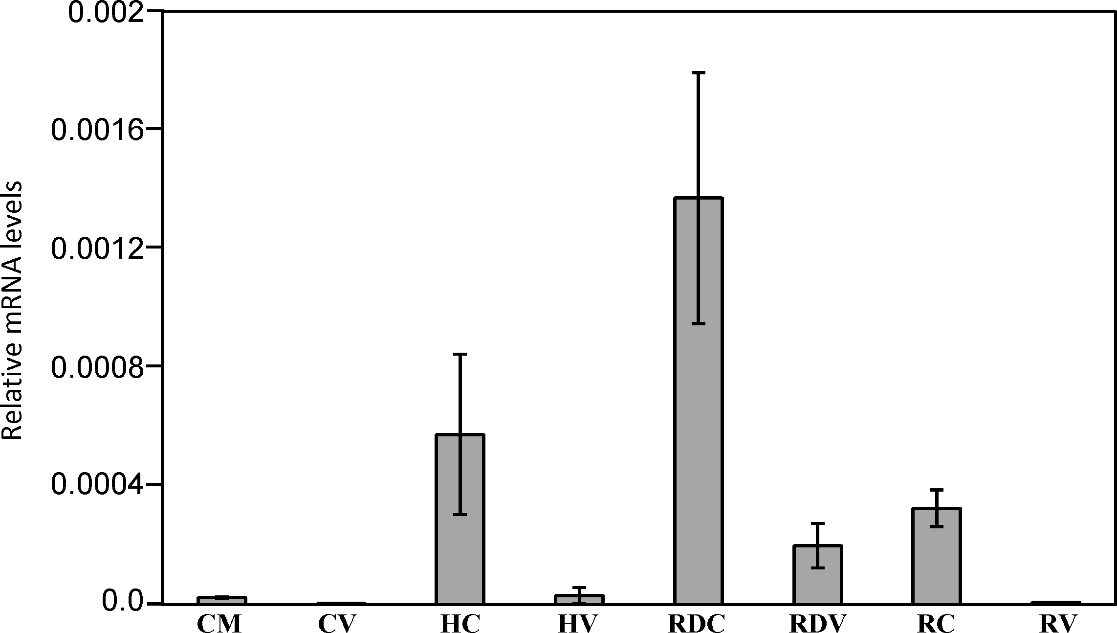


**Figure S1** Expression profile of *PpNAC1* in laser-microdissected tissues from one-month-old *P. pinaster* seedlings. The expression data were normalized using *Actin* as reference gene. Data are mean standard deviation from three biological replicates. CM, Cotyledon_mesophyll; CV, Cotyledon_vascular; HC, Hypocotyl_cortex; HV, Hypocotyl_vascular; RDC, Root_developing_cortex; RDV, Root_developing_vascular; RC, Root_cortex; RV, Root_vascular.

**Figure S2**





**Figure S2** Expression level of *PpMYB1*, *PpMYB4* and *PpMYB8* transcription factors in control (white) and *PpNAC1*_RNAi lines (mean of PN5 and PN9, gray) plantlets. Levels were analyzed by qPCR using *EF1-alpha* and *Actin* as standard gene for normalization. Data are mean standard deviation from three biological replicates. Asterisks indicate statistical differences between the *PpNAC1*_RNAi and control samples with a P-*value* < 0.05 by Student´s test. Error bars represent ± SD.

**Figure S3**





**Figure S3** Overexpression of *PpNAC1* in *Arabidopsis* plants. The full-length cDNA of *PpNAC1* driven by the CaMV 35S promoter was introduced into wild-type *Arabidopsis*. (a) Seedlings of overexpressors of *PpNAC1*_OE4.3 (middle) and *PpNAC1*_OE6.3 (right) showing curly leaves compared with the wild type (left). (b) Expression level of genes involved in the biosynthesis of cellulose (*CesA4, At5g44030;* *CesA7, At5g17420* and *CesA8, At4g18780*), xylan (*FRA8, At2g28110* and *IRX8, At5g54690*), and lignin (*CCoAOMT,* At4g34050 and *4CL1, At1g51680*) in seedling of 4-week-old *PpNAC1* overexpressors compared with the wild type (control). (c) Expression levels of secondary wall-associated transcription factors in the seedlings of *PpNAC1* overexpressors compared with the wild type. The Arabidopsis Genome Initiative locus identifiers fo the Arabidopsis genes investigated in this study are as follows: AtSND2 (At4g28500); AtNAC10 (At1g28470); AtNAC73 (At4g28500); MYB46 (At5g12870); MYB58 (At1g16490); MYB63 (At1g7918); MYB85 (At4g22680); KNAT7 (At1g62990). The expression level of each gene in the wild type is set to 1. Asterisks indicate statistical differences between overexpressors and control samples with a P-*value* < 0.05 by Student´s test. Error bars represent ± SD of three biological replicates.
