# Supplementary material for: PpNAC1, a main regulator of phenylalanine biosynthesis and utilization in maritime pine
Source: Plant Biotechnol J. 2017 Nov 23;16(5):1094–104. doi: 10.1111/pbi.12854 (PMC5902770; doi:10.1111/pbi.12854)
Supplement: Supplementary file 2 — Table S1 Oligonucleotides used in this work. [file PBI-16-1094-s003.pdf]

Table S1. Oligonucleotides used in this work.

Name	Sequence
**Cloning**	
Fw1 NAC1	5´- ATGACTTTATCAGTAAATGGGC- 3´
Rev1 NAC1	5´- TTACTTTACGAAGTTCCATAAA-3´
Rev2 NAC1	5´- CTCCTCCGTTGGATGGAACCGAAAGCC-3´
Rev3 NAC1	5´- CCTTGAAAGTTCCTGGAAACTGAAGCG-3´
Fw2 Pm NAC1	5´- GGCAGTTTAGGCAATTCCTTGGC- 3´
Rev Pm4 NAC1	5´- CTGACAACGAAGCACTGACCGGGAACT- 3´
Rev Pm5 NAC1	5´- TGACCGGGAACTGTCTGGACTCGAGAA- 3´
Rev Pm Myb8	5´- CTGAGATTCTGTTTGTAGCAG- 3´
Rev Pm Myb4	5´- CAGAGGCCTTTCCTTAGCTTGG-3´
**Expression analysis**	
FwqPCR NAC1	5´-CCGCAGCTTCGAATTAACAG- 3´
RevqPCR NAC1	5´-TGGAATTTGTTCTTAGGAGC- 3´
FwqPCR EF1α	5´- TGCTGTTGGAGTCATCAAGG-3´
RevqPCR EF1α	5´- CTCGTGCATCAGAATCAGACA-3´
FwqPCR Act	5´- ATCTCTCAGCACATTCCAACAG- 3´
RevqPCR Act	5´- TATTGCCACCATCATCTCAAGC- 3´
**RNAi construct**	
Fw NAC1_RNAi	5´-AAAAAGCAGGCTTAGCCATGGGATATTCAAGAG- 3´
Rev NAC1_RNAi	5´-AGAAAGCTGGGTCGCTGATGCGTGTAGTGCAAC- 3´
attB1	5´- ACAAGTTTGTACAAAAAGCAGGCT- 3´
attB2	5´- ACCACTTTGTACAAGAAAGCTGGGT- 3´
**Microarray validation**	
Fw spv3_21662	5´-CGGTCTGAACATGGACGAAG- 3´
Rev spv3_21662	5´- CACTTAAGTACGGCTGCGTC- 3´
Fw spv3_209693	5´- GGTGCTTCGGAGAATGAGAT-3´
Rev spv3_209693	5´- ATCCTTTGCTGCAGTCTTCC- 3´
Fw spv3_36713	5´- CATGTCTGCCTTAGCTGCAG- 3´
Rev spv3_36713	5´- ATGGTGACTTTCTTGGGTGG- 3´
Fw spv3_34116	5´- GCATATCCTGCGTCTCCTGC- 3´
Rev spv3_34116	5´- CTCCAGAAACCTCTGCCGCT- 3´
Fw spv3_210242	5´- CTGTTTGGATTCGGTCCAGG- 3´
Rev spv3_210242	5´- TAAGTACATGGCAACGAGGC- 3´
Fw spv3_10578	5´- GCTGGCCTCCATTTTCTCTC- 3´
Rev spv3_10578	5´- GAGGTAGCTAGCAGTCAACAC- 3´
Fw spv3_19461	5´- GATGGGCGGACAACTGTTTC- 3´
Rev spv3_19461	5´- TCATTCGGCGCTGGCTTTC- 3´
Fw spv3_3120	5´- GCGTCACCGCCAATGGGAAT- 3´
Rev spv3_3120	5´- CAGCGACAGCTTCTCTCCGT- 3´
Fw spv3_16431	5´- GTGGATGTGGAGGATTTGGC- 3´
Rev spv3_16431	5´- AGGAGATCACTACGAAGGGC- 3´
Fw spv3_26955	5´- GCAGTGGTGCTCGCATTATGG- 3´
Rev spv3_26955	5´- CACTGCAGATGCTCCACCAC- 3´
Fw qPCR 40S	5´- TCTTGAGAGTGGAGAATGGG- 3´
Rev qPCR 40S	5´- CGCATCAGTCATACTCACCT- 3´
**EMSA and transient expression analysis**	
Fw4 promoNAC1	5´- [BIOT]GTGAGATTCCAAAAACCCAAC- 3´
Rev2 promoNAC1	5´- [BIOT]CAATAACCTGAATCTACATTC- 3´
Fw promoMyb4	5´-[BIOT]GAGAAGGGATTGATTAACTTAAGAGTGAAGAAA

	CATATGGACAGATAGATTAG- 3´
Rev promoMyb4	5´- [BIOT]CTAATCTATCTGTCCATATGTTTCTTCACTCTTA AGTTAATCAATCCCTTCTC- 3´
Fw1 promoMyb8	5´- [BIOT]CCACTGAAGCAGCCCTTACAC- 3´
Fw2 promoMyb8	5´- [BIOT]GCACTACGACGAGTCCACGT- 3´
Rev1 promoMyb8	5´- [BIOT]CATAGTTAATTGCACTCCAAGG- 3´
Rev2 promoMyb8	5´- [BIOT]GTGGAGTTTCTCGTTTGTGG- 3´
Fw qPCR GUS	5´- GGTCGTCATGAAGATGCGGA- 3´
Rev qPCRGUS	5´- ATGCGAGGTACGGTAGGAGT- 3´
**Expression analysis of PpNAC1 overexpressing Arabidopsis**	
Fw CesA4	5´- TCAACATGGTCGGTGTTGTT- 3´
Rev CesA4	5´- GTCGGTGTTCGGTTTTGTCT- 3´
Fw CesA7	5´- TTGTTGCAGGCATCTCAGATG- 3´
Rev CesA7	5´- GCAGTTGATGCCACACTTGGA- 3´
Fw CesA8	5´- CGTTTTCCAAGGATTCCTCA- 3´
Rev CesA8	5´- AGCAACAACTCCGACCAAGT- 3´
Fw FRA8	5´- GACTTGTTGAATCGGTGGCTC- 3´
Rev FRA8	5´- ATTAGTAGCCGCCACGTGTT- 3´
Fw IRX8	5´- GCTATGCCGATGCTGAAAGT- 3´
Rev IRX8	5´- TCTCTGGGTGCCAATGTAGA- 3´
Fw CCoAOMT	5´- TCGTTGATGCTGACAAAGACA- 3´
Rev CCoAOMT	5´- ACTGATCCGACGGCAGATAG- 3´
Fw 4CL1	5´- TGGATTGATCGATGACGATG- 3´
Rev 4CL1	5´- GAACTTACGCGACAACAGCA- 3´
Fw AtNAC073	5´- AGGACCGACCTTACCTCCAC- 3´
Rev AtNAC073	5´- CCGGTCTGATTGAACGAGAT- 3´
Fw AtNAC010	5´- AAATGGAGGTGCTTTGTTGG- 3´
Rev AtNAC010	5´- TTAAAGTTACGCCCCCAATTT- 3´
Fw AtSND2	5´- AGGACCGACCTTACCTCCAC- 3´
Rev AtSND2	5´- CCGGTCTGATTGAACGAGAT- 3´
Fw AtMYB46	5´- GGAGTAAACGCAGGGGTACA- 3´
Rev AtMYB46	5´- TGGTCAAGGTCCCAAAATTC- 3´
Fw AtMYB58	5´- CAACAGCATAAACAGGGAACAG- 3´
Rev AtMYB58	5´- ATGCGCCTATCTGATCTTGC- 3´
Fw AtMYB63	5´- CAGGCTCAAGAGCAACAACA- 3´
Rev AtMYB63	5´- TGAGCTCGTAGTTCTTCAAGAGTG- 3´
Fw AtMYB85	5´- GATGAGGCCTCTTGGAGTGA- 3´
Rev AtMYB85	5´- CCCAAAATCATGAACACCAA- 3´
Fw EF1α	5´- TGAGCACGCTCTTCTTGCTTTCA- 3´
Rev EF1α	5´- GGTGGTGGCATCCATCTTGTTACA- 3´
